# Utilization of a human Liver Tissue Chip for drug-metabolizing enzyme induction studies of perpetrator and victim drugs

**DOI:** 10.1124/dmd.124.001497

**Published:** 2024-11-22

**Authors:** Shivam Ohri, Paarth Parekh, Lauren Nichols, Shiny Amala Priya Rajan, Murat Cirit

**Affiliations:** Javelin Biotech, Inc, Woburn, Massachusetts

**Keywords:** Cytochrome P450 (CYP), Drug-drug interactions, In vitro-in-vivo scaling, Pharmacokinetics, Liver microphysiological system

## Abstract

Polypharmacy-related drug-drug interactions (DDIs) are a significant and growing healthcare concern. An increasing number of therapeutic drugs on the market underscores the necessity to accurately assess new drug combinations during preclinical evaluation for DDIs. In vitro primary human hepatocytes (PHH) models are only applicable for short-term induction studies because of their rapid loss of metabolic function. Though coculturing nonhuman stromal cells with PHH has been shown to stabilize metabolic activity long-term, there are concerns about human specificity for accurate clinical assessment. In this study, we demonstrated a PHH-only liver microphysiological system in the Liver Tissue Chip is capable of maintaining long-term functional and metabolic activity of PHH from 3 individual donors and thus a suitable platform for long-term DDI induction studies. The responses to rifampicin induction of 3 PHH donors were assessed using cytochrome P450 activity and mRNA changes. Additionally, victim pharmacokinetic studies were conducted with midazolam (high clearance) and alprazolam (low clearance) following perpetrator drug treatment, rifampicin-mediated induction, which resulted in a 2-fold and a 2.6-fold increase in midazolam and alprazolam intrinsic clearance values, respectively, compared with the untreated liver microphysiological system. We also investigated the induction effects of different dosing regimens of the perpetrator drug (rifampicin) on cytochrome P450 activity levels, showing minimal variation in the intrinsic clearance of the victim drug (midazolam). This study illustrates the utility of the Liver Tissue Chip for in vitro liver-specific DDI induction studies, providing a translational experimental system to predict clinical clearance values of both perpetrator and victim drugs.

**Significance Statement:**

This study demonstrated the utility of the Liver Tissue Chip with a primary human hepatocyte–only liver microphysiological system for drug-drug interaction induction studies. This unique in vitro system with continuous recirculation maintains long-term functionality and metabolic activity for up to 4 weeks, enabling the study of perpetrator and victim drug pharmacokinetics, quantification of drug-induced cytochrome P450 mRNA and activity levels, investigation of patient variability, and ultimately clinical predictions.

## 1 Introduction

Drug-drug interactions (DDIs) can occur when multiple drugs are administered simultaneously, referred to as polypharmacy, leading to adverse drug reactions (Pirmohamed et al, 2004) with profound clinical effects that can reduce therapeutic efficacy or increase drug toxicity. This is a substantial healthcare burden because 37% of older adults use 5 or more drugs (Gu et al, 2010) that could potentially lead to DDI-related hospital admissions (Dechanont et al, 2014).

A therapeutic drug may lead to pharmacokinetic (PK) DDI either as a victim, where other drugs affect the investigational drug, or as a perpetrator, where the investigational drug affects concomitant drugs. Because most small-molecule drugs undergo biotransformation by cytochrome P450 (CYP) enzymes in the liver, CYP-mediated DDIs pose significant risk (Bohnert et al, 2016) and can result in several hundred-fold variations in victim drug exposure in humans (Backman et al, 1998). Thus, the evaluation of DDI risk of new investigational drugs is an essential step prior to clinical first-in-human studies.

Primary human hepatocytes (PHH) are considered the gold standard for metabolism-based in vitro studies; however, a major limitation is the loss of metabolic function over time (Fowler et al, 2020). This limitation challenges long-term drug incubations for perpetrator treatment as well as PK assessments of victim drugs (Lu and Di, 2020). To overcome this limitation, multiple in vitro coculture models were developed that cultured PHH with nonhuman stromal cells or liver nonparenchymal cell lines to extend the culture life of plated PHH (Hultman et al, 2016; Chan et al, 2019). However, the presence of stromal cell-like fibroblasts, either from nonhuman species or undefined cell type, complicates clearance predictions and can interact with the drugs in a nonphysiological manner (Kratochwil et al, 2017), highlighting the importance of the appropriate selection of supporting cell type for coculture with PHH, retaining physiological cytoarchitecture and interaction with the drugs. In addition to in vitro models, preclinical animal models were considered a viable option for metabolism-related DDI assessments (Prueksaritanont et al, 2006); however, they are rarely used in preclinical DDI studies as differences in species-specific CYP enzyme levels and tissue distribution make animal models less predictive of human enzyme induction (Lu and Li, 2001). Therefore, there remains a clear need for a metabolically active and human-specific in vitro system for preclinical DDI studies that enables long-term PK assessment of compounds and offers flexibility for testing various dosing regimens.

Human tissue chips, or microphysiological systems (MPSs), combine human cells, biomaterials, and biomimetic cues, such as fluid flow, to create physiologically relevant in vitro models with sustained metabolic activity. However, current microfluidic-based tissue chips face several challenges, including limited drug exposure in flow-through formats for studying low-clearance drugs, the use of drug-absorbing chip materials, small medium volumes, and tissue sizes for assays, and evaporation during long-term PK studies (Fowler et al, 2020). We have addressed these pitfalls with the development of a novel millifluidic system, Liver Tissue Chip (LTC), as described in our previous publication (Rajan et al, 2023). Here, we evaluated the LTC system for in vitro induction studies for multiple PHH donors. The LTC system was further evaluated for CYP-mediated DDI studies by inducing the system with rifampicin, a perpetrator drug, and conducting victim drug PK assessments with the known clinical victim drugs, midazolam and alprazolam.

## 2 Methods

### 2.1 Javelin’s LTC system

**Fig. 1:**
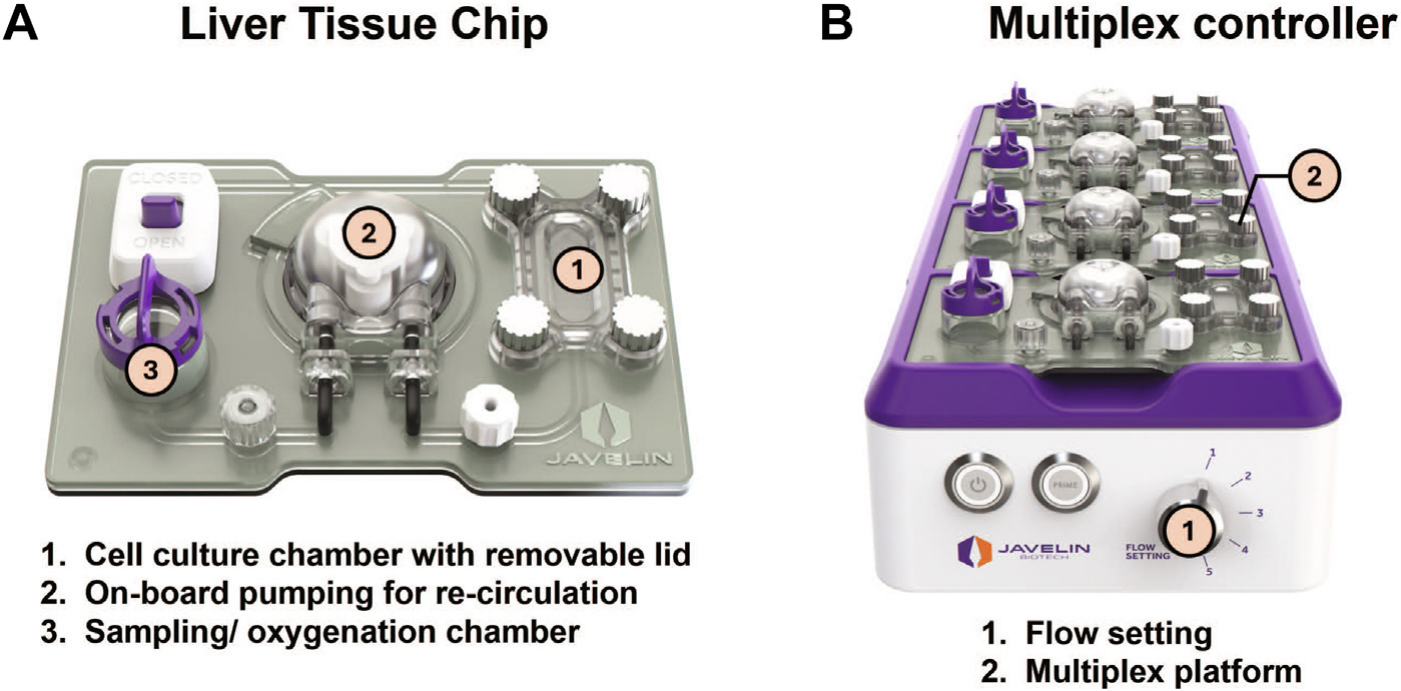
Human LTC system. The LTC system includes (A) LTC with key design features that enable long—term hepatic culture and (B) Multiplex controller that controls the medium recirculation of up to 4 connected LTCs at a predetermined flow rate.

The LTC system from Javelin Biotech, Inc. used in this study includes gamma-sterilized chips (Fig. 1A) and multiplex controllers (Fig. 1B). The basic working principles were described previously by Rajan et al (2023). Briefly, the thermoplastic-based LTC houses a 1-cm^2^ cell culture chamber with a removable sealing lid that allows for direct seeding into the chamber and access to the culture throughout the study. The on-board pump, controlled by the multiplex controller, allows for medium recirculation during the study at a set flow rate of 2 mL/h. The oxygenation chamber allows for continuous oxygen diffusion into the medium through a gas-permeable membrane, providing a constant oxygen supply to the cells. This chamber also allows for aseptic medium sampling for multiple kinetic time points during long-term PK studies.

### 2.2 Liver MPS culture and maintenance

#### 2.2.1 PHH culture

The liver MPS was comprised of PHH in sandwich culture format within the LTC culture chamber. Before cell seeding, the culture chamber was coated with an extracellular matrix solution consisting of 100 *μ*g/mL collagen (Corning) and 25 *μ*g/mL fibronectin (Sigma-Aldrich) prepared in phosphate-buffered saline, followed by overnight incubation at 4 °C and 2-hour incubation at 37 °C.

Cryopreserved PHHs were purchased from LifeNet Health (Donor 2214423, Female), AnaBios Corporation (Donor 1045, Female), and Thermo Fisher Scientific (Donor Hu8339, Female). Donor information including age, ethnicity, and cause of death is provided in (Supplemental Table 1). Cryopreserved PHH vials were thawed at 37 °C for 2 minutes and the cell suspension was transferred to a prewarmed hepatocyte thawing medium (Gibco). This suspension was centrifuged at 100 × *g* for 8 minutes and then resuspended in a hepatocyte plating medium (Gibco) at a density of 0.86 million cells/mL. Each cell chamber was seeded with 215,000 cells and incubated overnight. The attached cells were overlayed with 0.35 mg/mL Matrigel (Corning) prepared in an ice-cold hepatocyte maintenance medium (Gibco). Following overnight incubation, the cell chamber was sealed with the lid, and the chip was filled with 1.8 mL of maintenance medium and connected to flow for culture maintenance.

#### 2.2.2 Liver MPS maintenance

The liver MPS was maintained by replacing the spent medium every 4–7 days. The collected spent medium was used to run multiple biochemical assays quantifying albumin and urea production. The hepatocyte morphology was monitored every 2 days using bright-field microscopy (Zeiss).

### 2.3 Functional and metabolic activity assessment

Albumin production was quantified using an R-plex human albumin assay (Meso Scale Discovery), and urea synthesis was measured using a colorimetric assay (BioAssay Systems). Albumin and urea values were adjusted for total media volume and number of days elapsed between media changes and reported as *μ*g/day/million hepatocytes.

The liver MPS was incubated with a probe substrates cocktail for 5 major CYP isoforms (3 *μ*M midazolam, CYP3A4; 90 *μ*M diclofenac, CYP2C9; 3 *μ*M R-omeprazole, CYP2C19; 3 *μ*M tacrine, CYP1A2; 20 *μ*M dextromethorphan, CYP2D6) for 3 hours under recirculation at 37 °C and metabolism was assessed by metabolite quantification of 1-OH midazolam, 4-OH diclofenac, 5-OH omeprazole, OH-tacrine, and dextrorphan, respectively, using liquid chromatography-tandem mass spectrometry (see Bioanalysis in Supplemental Information; Supplemental Tables 2–6).

### 2.4 Real-time quantitative polymerase chain reaction

For real-time quantitative polymerase chain reaction (qPCR) analysis, RNA was extracted from the liver MPS using a guanidine thiocyanate-containing lysis buffer (Invitrogen) and further purified per the vendor protocol (Invitrogen). The total extracted RNA was converted to cDNA (Applied Biosystems), loaded onto custom Taqman array plates (Thermo Fisher Scientific), and run with the StepOnePlus Real-Time PCR system (Thermo Fisher Scientific). The fluorescence emitted during the amplification process was measured at each cycle, and the cycle threshold (Ct) values were determined. Relative gene expression levels were calculated using the 2^−ΔΔCt^ method with normalization to 2 reference genes, 18S and ACTB. The ΔCt was calculated by subtracting the Ct value from the control samples from the Ct value of the experimental group, and ΔΔCt was obtained by comparing the ΔCt of each target gene to that of the reference genes.

### 2.5 Liver MPS induction using rifampicin

The liver MPS was dosed with rifampicin for 72 hours to investigate the effects of 3 rifampicin dosing regimens: (1) daily spiking, (2) daily fresh dose, and (3) single dose. On day 3, using the complete medium change protocol, 1.8 mL of fresh medium containing 10 *μ*M rifampicin was added for all 3-dosing regimens. For the daily spiking regimen, the medium in the sampling chamber was spiked with 1.8 *μ*L of 10 mM rifampicin stock every 24 hours after the initial dose for an effective concentration of at least 10 *μ*M. In the daily fresh dose condition, the spent rifampicin-dosed medium was replaced every 24 hours with a fresh medium containing 10 *μ*M rifampicin until 72 hours. For the single-dose regimen, no drug spiking or medium replacement was conducted throughout the 72 hours of induction before treatment. At the end of rifampicin incubation, metabolic activity and/or mRNA changes in the liver MPS were evaluated using probe substrate cocktail assay and qPCR analysis, respectively.

### 2.6 Long-term PK assessment with victim drugs

Long-term PK of the victim drugs, midazolam and alprazolam, was assessed after treatment with the perpetrator drug, rifampicin. The substrates midazolam (1 *μ*M) or alprazolam (1 *μ*M) were added to the liver MPS as a bolus dose on day 6 in the presence and absence of 10 *μ*M rifampicin. Medium samples of 50 *μ*L were collected from the sampling chamber every 24 hours for 72 and 120 hours for midazolam and alprazolam, respectively. Samples were analyzed for parent drug depletion and metabolite formation using liquid chromatography-tandem mass spectrometry (see Bioanalysis in Supplemental Information). Area under the curve (AUC) of the substrate (victim drug) depletion curve was calculated using GraphPad Prism (GraphPad Software, Inc) and was represented as AUC _0–t_ where ‘t’ represents the endpoint of the victim drug study. In vitro intrinsic clearance (CL_int_ [*μ*L/min/10^6^ hepatocytes]) was calculated using the slope of substrate depletion data and predicted in vivo intrinsic clearance (CL_h_ [mL/min/kg]) was estimated after physiological scaling of human factors. Equations and experimental values used for scaling in vitro parameters are previously described in the study by Rajan et al (2023). Briefly, unbound in vitro intrinsic clearance (CL_int(u)_) was calculated by dividing CL_int_ by the fraction unbound in the media (fu_media_), which was scaled to human liver equivalent unbound intrinsic clearance (CL_int(u),__H_ [mL/min/kg]) taking into account human hepatocellularity and liver weight. Finally, the upscaled human CL_int(u)_ was converted to predicted human hepatic clearance (CL_h_ [mL/min/kg]) using well stirred (WS) and parallel tube (PT) models.

### 2.7 Drug *stock preparations*

Drugs were purchased in powdered form from Sigma-Aldrich (rifampicin, diclofenac, and dextromethorphan), Cayman Chemicals (R-omeprazole), and Tocris (tacrine). Drugs were dissolved in dimethyl sulfoxide (DMSO) to prepare stock solutions at a concentration at least 1000 times higher than the required final concentration; stock solutions were stored at −80 °C until use. Midazolam and alprazolam (Sigma-Aldrich) were purchased presolubilized in methanol; 1 mM stock solutions were prepared in DMSO and diluted to a final concentration of 1 *μ*M in the medium. All drug incubations in the LTC were conducted with a DMSO volume/volume percentage of <0.5.

### 2.8 Statistical analysis

To determine statistical significance, unequal variance *t* tests were performed between 2 conditions, and the level of significance was determined using *P* value ranges, with *P* < .05 deemed significant. A minimum of 3 independent biological replicates were performed for each experimental group. Microsoft Excel and GraphPad Prism were used for data analysis and GraphPad Prism was used to plot the data.

## 3 Results

### 3.1 Long-term maintenance of liver MPS from multiple PHH donors

**Fig. 2:**
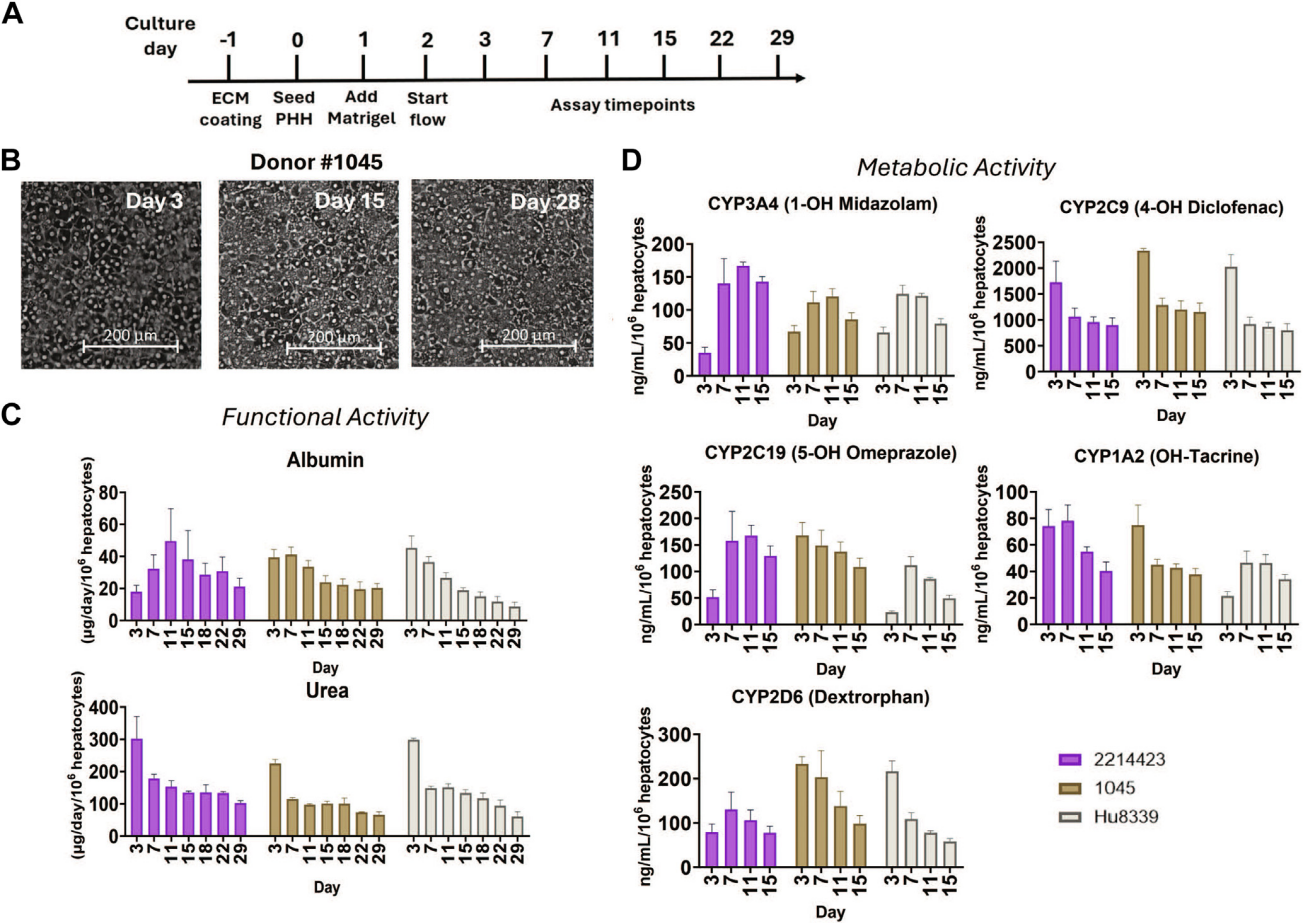
Biological characterization of PHH from 3 donors (2214423, 1045, and Hu8339) maintained in the LTC. (A) Optimized experimental workflow for long-term liver MPS maintenance in LTC. (B) Bright field images of PHH (donor 1045) showing tissue morphology that was maintained for at least 28 days. Liver MPS from all 3 donors sustained long-term. (C) functional activity quantified by measuring albumin and urea production until day 29 and (D) metabolic activity measured by incubating the liver MPS with 5-in-1 probe substrate cocktail for CYP3A4, CYP2C9, CYP2C19, CYP1A2, and CYP2D6 and quantifying associated metabolites until at least day 15. Plotted data represent mean ± SD of 3–4 biological replicates. ECM, extracellular matrix.

In the LTC system, PHH from 3 different donors (2214423, 1045, and Hu8339) were maintained in sandwich culture format for over 4 weeks using the optimized protocol (Fig. 2A). The functionality of the liver MPS was evaluated by measuring the production of albumin and urea while monitoring PHH morphology using bright-field imaging. PHH from all 3 donors maintained their cobblestone morphology (Fig. 2B and Supplemental Fig. 1) and prolonged production of albumin and urea throughout the culture period of 4 weeks (Fig. 2C). Healthy liver MPS produced an average albumin level per day ranging from ∼20–50 *μ*g/day/10^6^ hepatocytes during the culture duration, which is within physiological levels (Baudy et al, 2020). The PHH culture from Hu8339 showed donor variability toward the end of the culture period, where albumin levels were below 20 *μ*g/day/10^6^ hepatocytes after day 15. All donors displayed higher levels of urea production on day 3 of culture, followed by sustained production with average levels ranging from ∼100–300 *μ*g/day/10^6^ hepatocytes. The stable production of albumin, a protein synthesis marker, and urea, a metabolic function marker, demonstrates the maintenance of long-term healthy liver MPS with sustained hepatocyte phenotype and viability.

PHHs from all the donors sustained metabolic potential for at least 15 days, confirming metabolically active and stable liver MPS for DDI studies (Fig. 2D). CYP3A4 and CYP2C19 activity levels (measured by quantifying 1-OH midazolam and 5-OH omeprazole, respectively) showed an increase for all 3 donors from day 3 of the culture period, with sustained activity from day 7 except for CYP2C19 activity of 1045, which showed sustained activity levels from day 3 onward. In contrast, activity levels of CYP1A2, CYP2C9, and CYP2D6 (quantified by OH-tacrine, 4-OH diclofenac, and dextrorphan, respectively) showed higher activity on day 3, which is a day after flow, followed by sustained activity during the culture period. Donor variability was observed for CYP1A2 activity levels in Hu8339 and CYP2D6 activity levels in 2214423, where an increase in enzyme activity levels was observed after day 3 for both the donors, with activity levels stable in later days of culture. Additionally, we also quantified the metabolic activity of 2 donors, 2214423 and 1045, up until 29 days, showing the capability of the LTC system to perform metabolism-based studies beyond 2 weeks (Supplemental Fig. 2). This demonstrates the ability of LTC to preserve the long-term CYP activity of PHH from multiple donors while capturing their interdonor differences, making it a well suited in vitro platform for conducting metabolic studies and achieving more accurate predictions.

### 3.2 Effects of different rifampicin dosing regimens on CYP activity levels

**Fig. 3:**
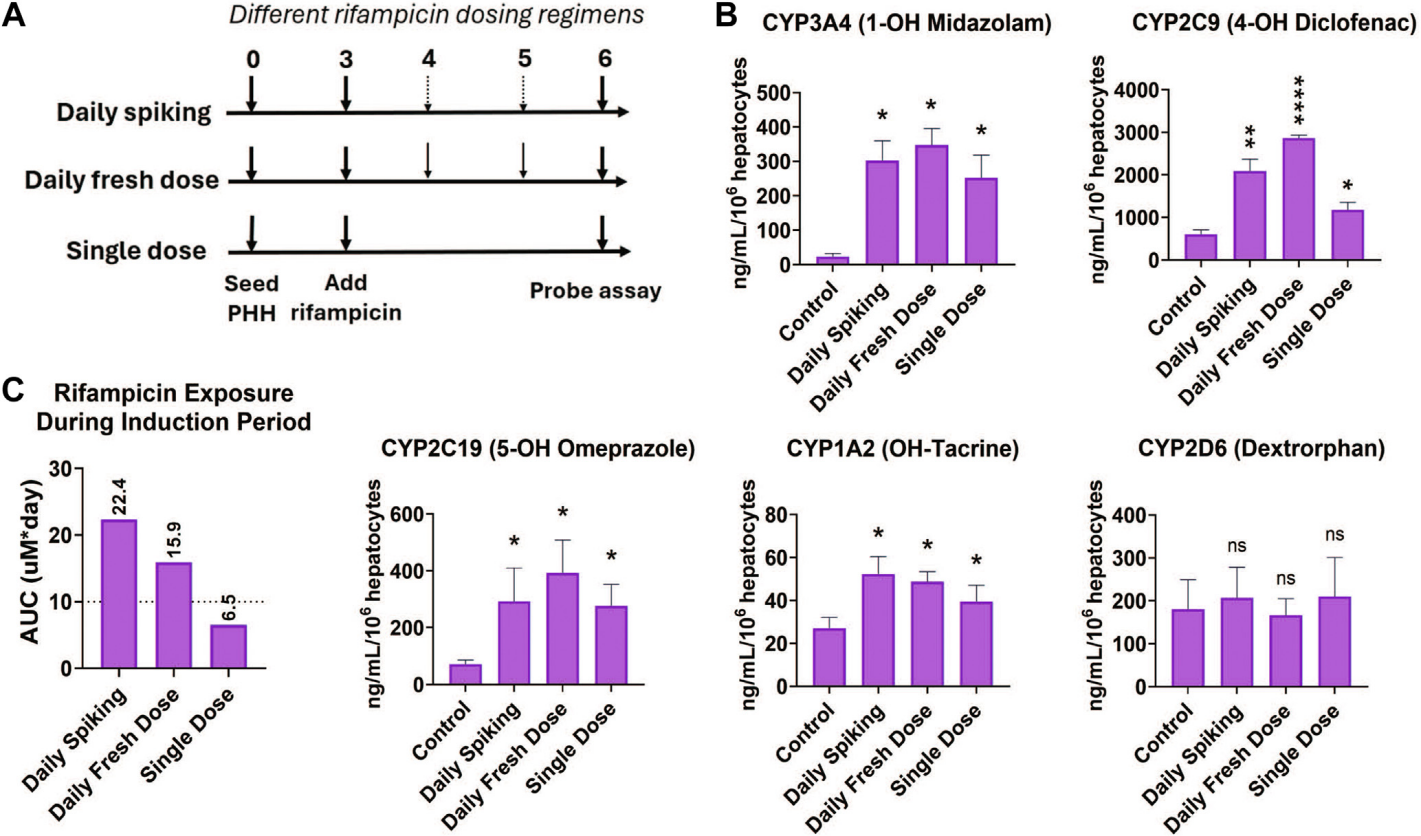
Investigating induction effects of rifampicin on LTC across different rifampicin dosing regimens (PHH donor: 1045). (A) Experimental workflow of dosing regimens: daily spiking, daily fresh dose, and single dose. (B) Activity levels of CYP3A4, CYP2C9, CYP2C19, CYP1A2, and CYP2D6 enzymes were quantified using 5-in-1 probe substrate cocktail assays after rifampicin treatment across different dosing regimens. Compared with the vehicle control, the liver MPS showed dose regime–dependent variation only for CYP2C9 levels and no significant differences among regimens for other measured CYP enzymes (significance level not shown). (C) shows the rifampicin exposure to liver MPS for different dosing regimens represented as AUC of rifampicin concentration-time curve obtained during 3 days of the induction pretreatment period. Statistical significance is displayed relative to control determined using unequal variance *t* test (∗.001 < *P* < .05; ∗∗.0001 < *P* < .001; ∗∗∗.00001 < *P* < .0001; ∗∗∗∗*P* < .00001; no significance (ns) *P* > .05). Plotted data represent the mean ± SD of 3–4 biological replicates.

The liver MPS (donor 1045) was induced with rifampicin using 3 dosing regimens over 72 hours: (1) daily spiking, (2) daily fresh dose, and (3) single dose (Fig. 3A and Supplemental Fig. 3A). All 3 rifampicin dosing regimens showed similar levels of induced CYP3A4, CYP2C19, and CYP1A2 activities in LTC. Interestingly, the daily fresh regimen resulted in significantly higher CYP2C9 activity than the other 2 regimens (Fig. 3B). For each dosing regimen, the rifampicin concentration in LTC was quantified daily during the induction period between days 4 and 6 (Supplemental Fig. 3, B–D). Rifampicin exposure was calculated by quantifying the AUC of the concentration over 72 hours of induction before treatment. The AUC results revealed the highest rifampicin exposure of 22.4 *μ*M∗day for the daily spiking regimen, 15.9 *μ*M∗day with the fresh daily dose, and the lowest exposure of 6.6 *μ*M∗day for the single rifampicin dose (Fig. 3C). Based on the highest rifampicin exposure, the daily spiking regimen was used for subsequent studies.

### 3.3 Evaluation of donor variability in rifampicin-induced CYP mRNA expression and activity levels

**Fig. 4:**
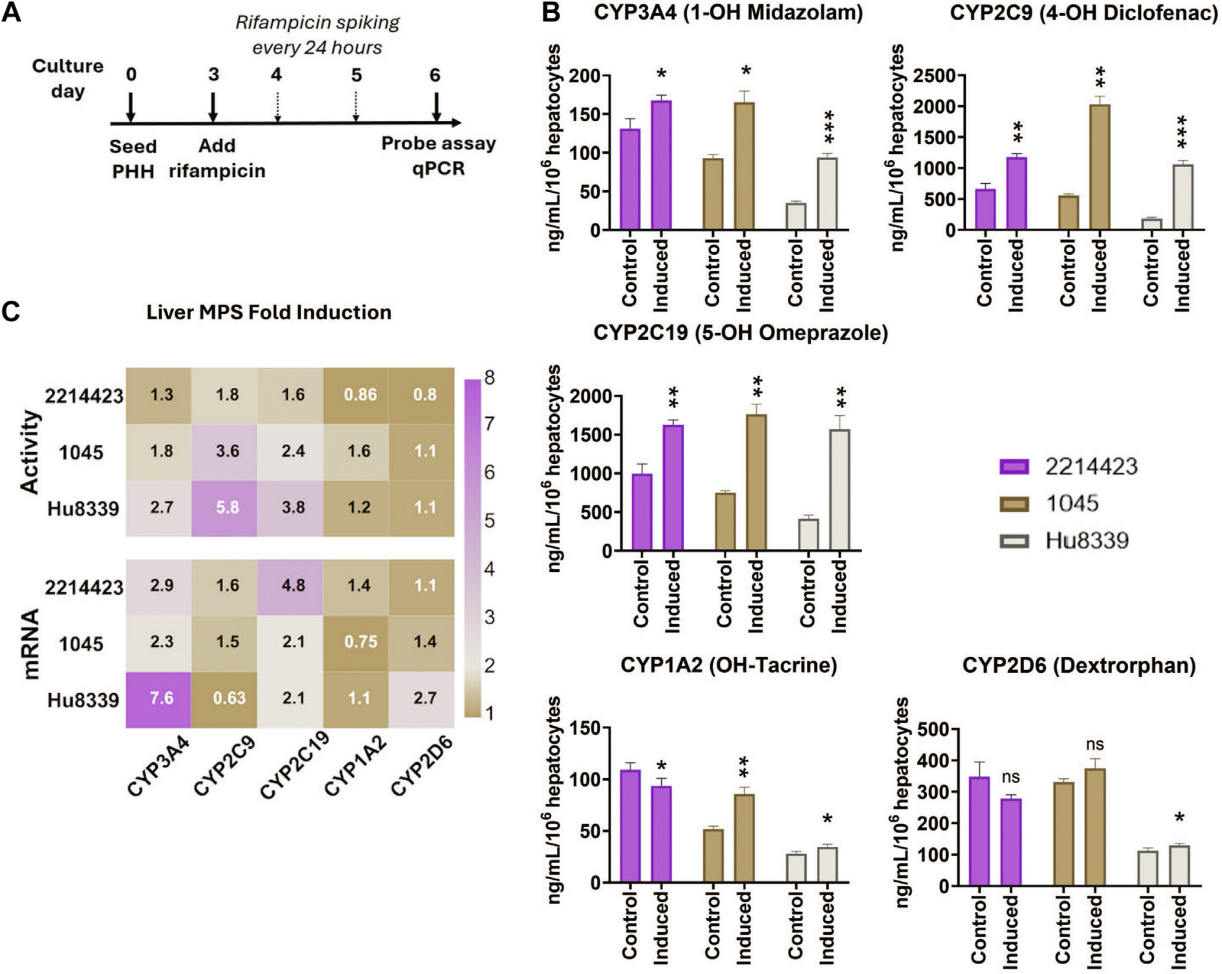
Comparison of rifampicin-mediated induction effects on liver MPS across different donors (2214423, 1045, and Hu8339) (A) Experimental workflow with optimized daily spiking dosing regimen. (B) Enzyme activity levels of CYP3A4, CYP2C9, CYP2C19, CYP1A2, and CYP2D6 quantified using 5-in-1 probe substrate cocktail assay show rifampicin-mediated induction compared with the vehicle control for all 3 donors. (C) Fold induction changes for mRNA and enzyme activity levels across different donors demonstrate donor variability observed in induced levels of enzyme activity from control. Statistical significance is displayed relative to control determined using an unequal variance *t* test (∗.001 < *P* < .05; ∗∗.0001 < *P* < .001; ∗∗∗.00001 < *P* < .0001; ∗∗∗∗*P* < .00001; no significance (ns) *P* > .05). Plotted data represent the mean ± SD of 3–4 biological replicates.

Liver MPS from 3 donors were induced with rifampicin on day 3 for 72 hours using the daily spiking regimen (Fig. 4A). At the end of the induction period, CYP enzymatic activity was assessed using a 5-plex CYP probe cocktail, and CYP mRNA levels were assessed by qPCR. Rifampicin treatment induced CYP3A4, CYP2C9, and CYP2C19 activities for all donors compared with the vehicle control (Fig. 4, B and C). While CYP2D6 activity was not affected by rifampicin treatment with any of the donors, CYP1A2 induction was observed with one donor, 1045. CYP3A4 activity was induced 1.3-, 1.8-, and 2.7-fold for 2214423, 1045, and Hu8339, respectively, while fold increase of mRNA expression levels (from 2.3 to 7.6-fold increase) was higher than enzymatic activity levels from each donor (Fig. 4C). Interestingly, CYP2C9 mRNA expression after induction was <2-fold for each donor; however, the range of increase in the enzymatic activity was higher (1.8–5.8-fold) than the mRNA fold increase range (from 0.63- to 1.6-fold).

### 3.4 Baseline recovery of CYP activity levels after rifampicin withdrawal

**Fig. 5:**
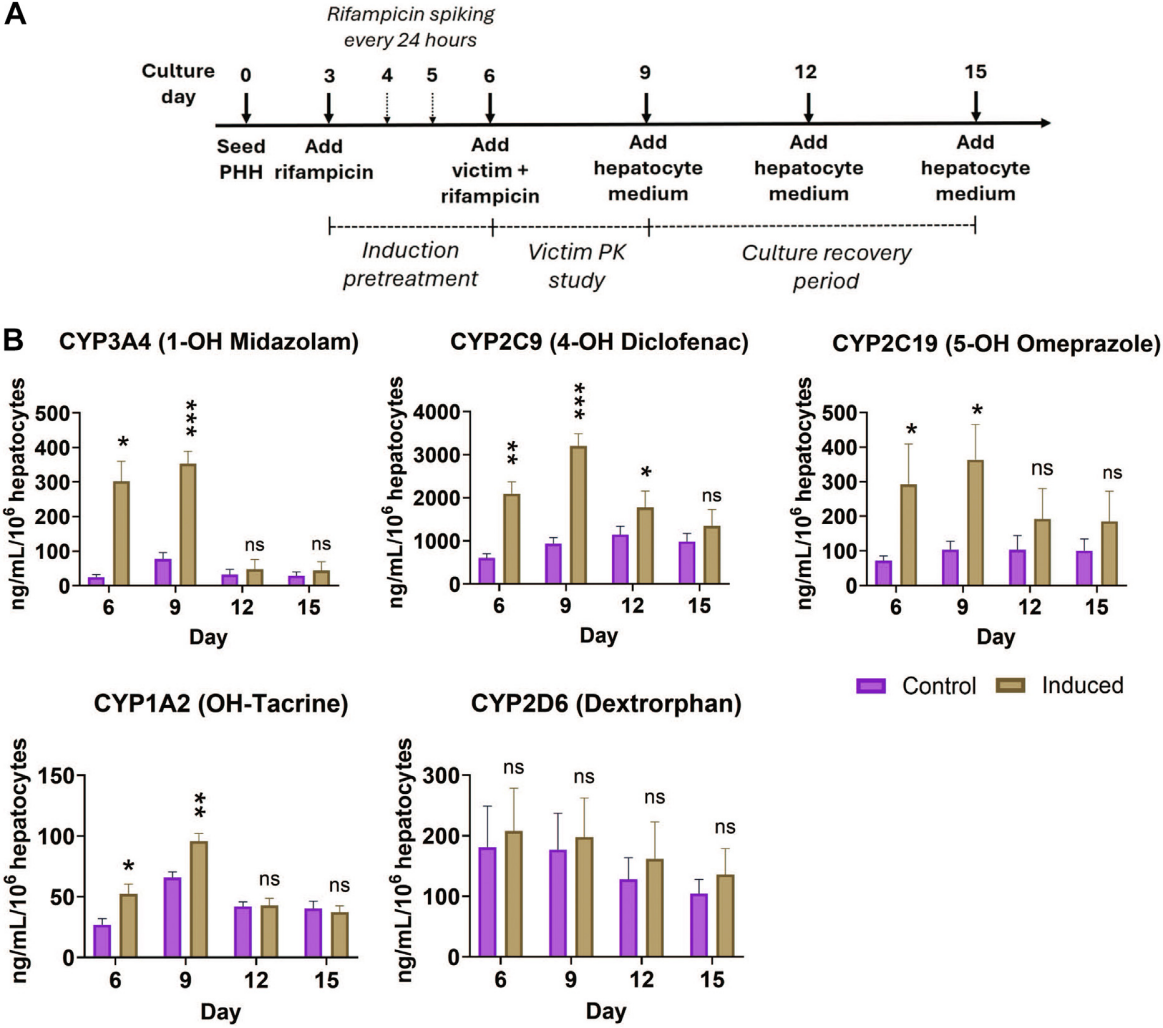
Time course and enzyme activity levels of the liver MPS during long-term drug-drug interaction study involving rifampicin—mediated induction, victim PK study, and deinduction after cessation of rifampicin (PHH donor: 1045). (A) Experimental workflow of DDI victim-perpetrator study with recovery time course. (B) Time course plots of CYP activity levels (CP3A4, CYP2C9, CYP2C19, CYP1A2, and CYP2D6) show increase in activity during rifampicin treatment (days 3–9) compared with the vehicle control followed by deinduction that is observed by activity levels returning to baseline levels after cessation of rifampicin treatment on day 9. Statistical significance is displayed relative to control determined using unequal variance *t* test (∗.001 < *P* < .05; ∗∗.0001 < *P* < .001; ∗∗∗.00001 < *P* < .0001; ∗∗∗∗*P* < .00001; no significance (ns) *P* > .05). Plotted data represent the mean ± SD of 3–4 biological replicates.

The liver MPS (donor 1045) was induced with rifampicin starting at day 3 for 72 hours using the daily spiking regimen (induction pretreatment period); at day 6, a single dose of 10 *μ*M rifampicin was added for another 72 hours (victim PK study period). On day 9, a rifampicin-free hepatocyte maintenance medium was added for an additional 6 days to observe the baseline recovery of CYP activity levels (Fig. 5A and Supplemental Fig. 4A). CYP activity levels were assessed on days 6, 9, 12, and 15. After the induction pretreatment period (day 6) and at the end of the victim PK study period (day 9), CYP3A4, CYP2C9, and CYP2C19 activities were maintained at the high levels, compared with the vehicle control, expected of an induced condition. After removing rifampicin on day 9, CYP3A4- and CYP2C19-induced activity returned to baseline levels within 3 days and CYP2C9 reduced within 6 days (Fig. 5B). Additionally, we characterized the effects of the daily fresh dose and single-dose regimens of rifampicin on CYP activity recovery. The single-dose regimen of rifampicin resulted in similar reduction kinetics of CYP3A4 and CYP2C19 activity as daily spiking. However, CYP2C9 activity returned to baseline levels within 3 days rather than the 6 days observed after daily spiking of rifampicin. The daily fresh dose regimen also resulted in similar CYP3A4 activity recovery as daily spiking; however, CYP2C9 and CYP2C19 activity did not completely return to baseline levels after induction pretreatment (Supplemental Fig. 4B).

### 3.5 Victim drug intrinsic clearance changes in response to a perpetrator

**Fig. 6:**
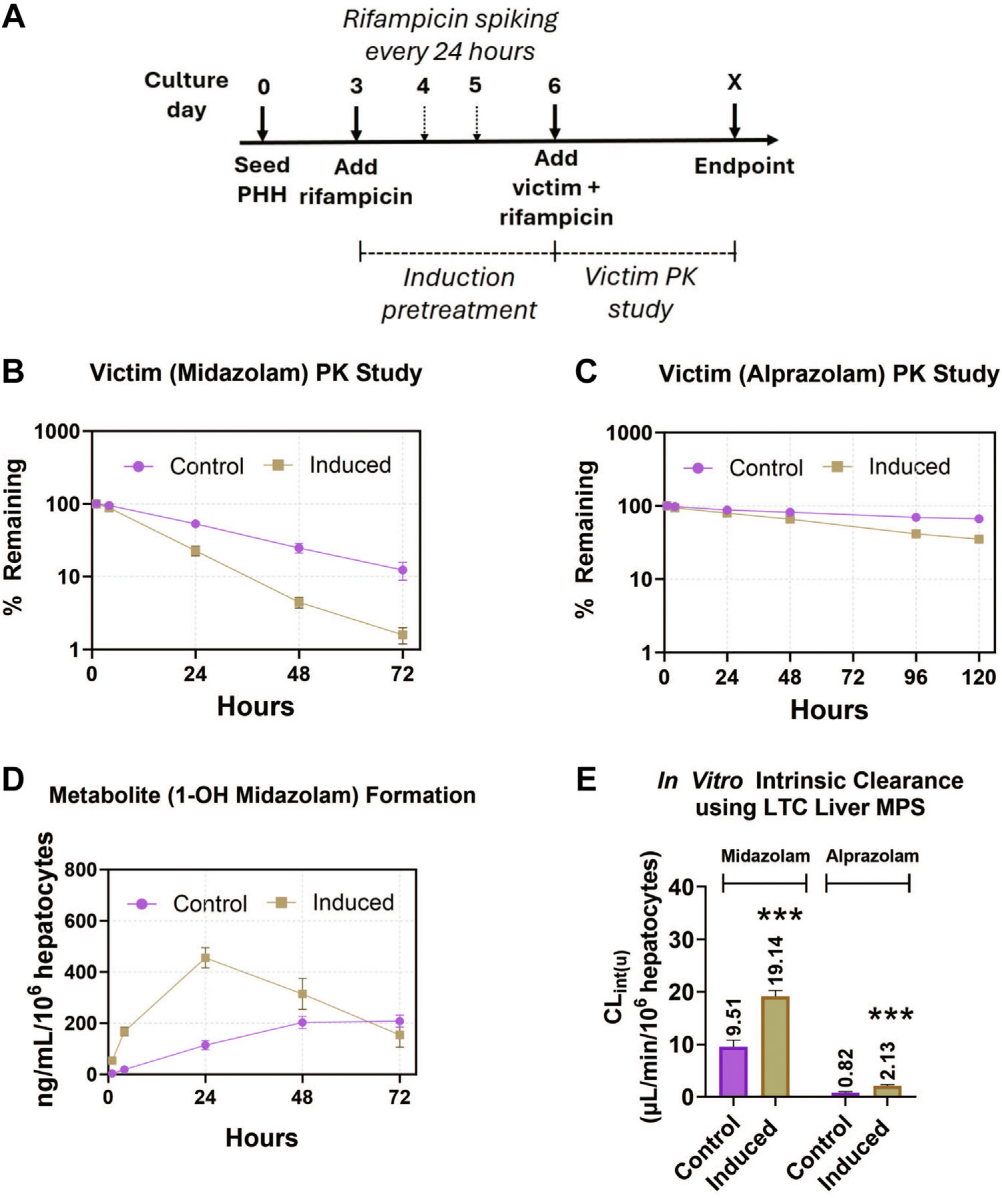
DDI observed in LTC during long-term victim-perpetrator study (PHH donor: 1045). (A) Experimental workflow of victim-perpetrator PK study with daily spiking of rifampicin regimen during induction pretreatment. The PK profile of both (B) high-turnover drug midazolam and (C) low-turnover drug alprazolam shows increased depletion rate in rifampicin—induced liver MPS compared with the vehicle control. (D) It shows increased production of primary metabolite (1-OH midazolam) of midazolam in induced liver MPS compared with the control. € Unbound in vitro intrinsic clearance of victim drugs from induced and controlled liver MPS. Statistical significance is displayed relative to control determined using an unequal variance *t* test (∗.001 < *P* < .05; ∗∗.0001 < *P* < .001; ∗∗∗.00001 < *P* < .0001; ∗∗∗∗*P* < .00001; no significance (ns) *P* > 0.05). Plotted data represent the mean ± SD of 3–4 biological replicates.

The liver MPS (donor 1045) was induced with rifampicin starting at day 3 for 72 hours; then, at day 9, 1 *μ*M victim drugs (midazolam or alprazolam) were codosed with 10 *μ*M rifampicin, the perpetrator drug, for victim PK studies (Fig. 6A and Supplemental Fig. 5A). Midazolam, a high clearance compound, and alprazolam, a low-clearance compound, are both mostly metabolized by CYP3A4 enzyme. We observed higher intrinsic clearance values for each drug in the rifampicin induction group compared with the untreated liver MPS. The midazolam victim study showed a reduction of 43% in the AUC_0–72hr_ of parent drug concentration compared with the control group (Fig. 6B). Unbound in vitro intrinsic clearance for the rifampicin-induced liver MPS (19.14 *μ*L/min/million cells) and vehicle control (9.51 *μ*L/min/million cells) showed a 2-fold increase in midazolam clearance (Fig. 6E). The predicted human hepatic clearance values of midazolam using the WS model and the PT model were 4.23 mL/min/kg and 4.67 mL/min/kg for the induced liver MPS and 2.34 mL/min/kg and 2.47 mL/min/kg for the control, respectively (Table 1). Daily fresh and single-dose regimens of rifampicin induction resulted in 54% and 42% reduction in the AUC_0–72hr_ of the midazolam depletion curve, with 2.2- and 1.98-fold increase in intrinsic clearance values, respectively (Supplemental Fig. 5, B and C), resulting in no significant differences between any of the 3 dosing regimens. Predicted human hepatic clearance values of midazolam from the liver MPS induced with different rifampicin dosing regimens also displayed similar results (Supplemental Table 7). The primary metabolite of midazolam (1-OH midazolam) was quantified to demonstrate inducibility and increased metabolite production in the presence of rifampicin. There was a 2.1-fold increase in the AUC of 1-OH midazolam formation rate from rifampicin-induced liver MPS compared with the vehicle control (Fig. 6D). For alprazolam, the AUC_0–120hr_ of the parent drug was reduced by 22% for the induced liver MPS (Fig. 6C), while the unbound intrinsic clearance values were increased from 0.82 *μ*L/min/million cells for vehicle control to 2.13 *μ*L/min/million cells for the induced liver MPS, resulting in a 2.6-fold increase (Fig. 6E). The predicted human hepatic clearance estimates of alprazolam were also calculated using the WS model and the PT model, with resulting values of 2.33 mL/min/kg and 2.46 mL/min/kg for the induced liver MPS and 0.96 mL/min/kg and 0.98 mL/min/kg for the vehicle control, respectively (Table 1).

**Table 1:** Predicted human CL_h_ values using WS and PT models CL_int(u)_ of the victim drugs (midazolam and alprazolam) were calculated for both rifampicin-induced and vehicle control using the LTC substrate depletion data, which was scaled to predicted human hepatic clearance values using human hepatocellularity and WS and PT models.

Drug	Condition	CL_int(u)_ (*μ*L/min/10^6^ hepatocytes)	CL_h_ (WS) (mL/min/kg)	CL_h_ (PT) (mL/min/kg)
Midazolam	Control	9.51	2.34	2.47
	Induced	19.14	4.23	4.67
Alprazolam	Control	0.82	0.96	0.98
	Induced	2.13	2.33	2.46

## 4 Discussion

Prediction of CYP-mediated DDI risk of a new investigational drug during the preclinical drug development process is an essential step before clinical studies. Such assessment requires an in vitro system capable of maintaining long-term hepatic metabolic activity of PHH to conduct intrinsic clearance estimation and enzyme induction studies (Fowler et al, 2020). To demonstrate the utility of LTC for in vitro DDI studies, we evaluated the system for long-term functional and metabolic activities of multiple PHH donors and performed rifampicin induction of multiple CYP isoforms to obtain clinically comparable results for victim drug intrinsic clearance changes in response to a perpetrator drug.

The LTC was biomimetically designed to maintain the PHH culture under low shear stress and in an oxygen-rich microenvironment (Rajan et al, 2023), which is known to improve and sustain functional and metabolic activity of hepatocytes (Kidambi et al, 2009). An optimal flow rate was determined in the previous study, which enables sufficient delivery of oxygen and nutrients to the cells while efficiently removing cellular waste from the cell culture chamber. PHH culture maintained under flow in the LTC showed higher albumin and urea production compared with sandwich culture maintained under static conditions (Rajan et al, 2023). In this study, we demonstrated LTC maintaining prolonged functional activity of healthy PHH from 3 different donors without the addition of supporting cells, producing physiologically relevant albumin levels. The observed albumin production range of 20–50 *μ*g/day/10^6^ hepatocytes is comparable to the existing long-term complex micropatterned coculture model consisting of PHH and nonhuman stromal cells (Khetani and Bhatia 2008), showing the novelty of the engineered millifluidic in vitro system to extend the functionality of plated PHH. Long-term metabolic activity was sustained across all donors, as demonstrated with multiple CYP isoforms. These results indicate the applicability of the LTC system for long-term PK studies. CYP1A2, CYP2D6, and CYP2C9 activity profiles showed similar trends of higher activity 1-day post introduction of flow-induced shear stress and sustained expression throughout, as opposed to CYP3A4 and CYP2C19 activity levels, where an increase in CYP activity was observed as the culture progressed. This could be due to the varying effects of fluidic flow on different CYP isoforms (Du et al, 2017).

Donor variability was observed for multiple CYP isoforms, which displayed different activity levels and profiles. Such variability in metabolic activity is also observed in the clinic and depends on multiple factors, including biological sex, age, and ethnicity, which can all impact clinical outcomes (Zanger and Schwab, 2013). Higher clearance of midazolam has been reported in South Asian compared with Caucasian populations (van Dyk et al, 2018), and women exhibited higher clearance of midazolam, a CYP3A substrate (Zhu et al, 2003). Thus, PHH-based liver MPS can be used as an in vitro research tool to study individual donors from various backgrounds to predict clinical profiles for special populations.

The inducibility of PHH from multiple donors was assessed by measuring both CYP mRNA and activity levels. We observed more consistent induction of mRNA levels than activity with the exception of CYP2C9, suggesting that mRNA is a more sensitive induction marker than activity (Fahmi et al, 2010). All 3 donors were induced >2-fold for CYP3A4 and CYP2C19 mRNA, which is above the threshold of a positive in vitro induction signal from treatment with rifampicin (Kenny et al, 2018). CYP3A4 activity fold induction levels did not reach greater than 2-fold for 2 donors, which could be due to high baseline activity levels of the donors indicating a stable and metabolically active healthy liver MPS. Nevertheless, CYP3A4, CYP2C9, and CYP2C19 activity levels of all 3 hepatocyte donors were increased significantly in response to rifampicin treatment. We observed experiment-to-experiment variability in induction levels for donor 1045, consistent with literature reporting wide fold induction ranges for the same PHH donor (Kenny et al, 2018). The ’LTC’s ability to maintain long-term PHH metabolic activity, unlike typical sandwich cultures, allowed us to study deinduction of CYP activity levels in the liver MPS after rifampicin withdrawal and compare it with the baseline control group. CYP3A4 activity levels returned to baseline within 3 days after being in the presence of rifampicin for 6 days, including induction pretreatment with multiple dosing regimens and the victim PK study conducted in the presence of rifampicin. Such in vitro studies monitoring long—term metabolic activity levels could provide insight into clinical CYP activity recovery after being induced, impacting victim drugs and potentially helping improve clinical trial designs (Imai et al, 2008; Reitman et al, 2011).

Different dosing regimens—daily spiking, daily fresh, and single doses—of the perpetrator drug, rifampicin, were investigated for their CYP induction potential. Daily spiking resulted in the highest exposure of rifampicin followed by a daily fresh dose and then single dose. Despite these different exposures, CYP3A4 activity was induced to the same levels with all dosing regimens, leading to no significant changes in midazolam intrinsic clearance compared with vehicle control. Such studies could provide insight into dosing regimens of perpetrator drugs and associated DDI, potentially incorporating multiple scenarios and enabling the selection of appropriate dosing strategies before clinical studies are conducted (Herman et al, 1983; Xu et al, 2011).

In vitro assessment of victim drug intrinsic clearance after induction is a challenge, especially for low-clearance compounds, because longer incubation times with metabolically active liver cultures are required (Di and Obach, 2015). Here, we demonstrated victim PK studies with both a low hepatic clearance compound, alprazolam, and a high hepatic clearance compound, midazolam. The intrinsic clearance of midazolam in the LTC increased 2-fold after induction, and the AUC_0–72hr_ of the midazolam depletion curve reduced by 43%. This is comparable to observed clinical results, where the AUC_0–∞_ of intravenously dosed midazolam was reduced by 55%, leading to a 2.18-fold increase in systemic clearance after rifampicin administration for 7 days (Gorski et al, 2003). Similar to midazolam, there was a 2.6-fold increase in alprazolam intrinsic clearance in rifampicin-induced LTC, with a reduction of 22% in AUC_0–120hr_ of alprazolam. In addition to long-term metabolically stable liver MPS, continuous recirculation in the LTC without any medium change during the victim PK study allowed for the accumulation of primary metabolite, 1-OH midazolam, generated from the parent drug, midazolam. Both parent drug and primary metabolite were quantified from the multi–time point collected samples for both induced and control LTC, displaying higher metabolite generation after induction. Such a design could also be applied to studies where the effects of generated metabolites need to be evaluated pre-clinically to avoid unexpected interactions in the clinic (Templeton et al, 2016; Steinbronn et al, 2021).

In this study, we focused on induction-based DDIs occurring at the hepatic level using a liver-only MPS system that supports the assessment of intravenously dosed drugs. However, oral administration is the most common drug administration route, and drugs having high intestinal metabolism are more susceptible to inducer-based DDI (Xie et al, 2016). For example, midazolam, which is mostly metabolized by CYP3A4, is metabolized almost equally in the liver and gut. Clinically, this leads to an AUC change of 98.5% in the plasma concentration-time curve of orally dosed midazolam after rifampicin treatment compared with only a 34.5% change for intravenously dosed midazolam after rifampicin treatment (Link et al, 2008). Thus, future work will include studying DDIs for drugs with both routes of administration: intravenously (ie, liver-only system) and oral (ie, liver and gut integrated system). Such an MPS platform would enable investigation of DDIs caused by induction at both hepatic and intestinal MPSs and evaluation of multiple PK parameters related to oral drug administration, such as clearance values in gut and liver MPS, gut MPS drug permeability, and bioavailability (Tsamandouras et al, 2017). PK parameters evaluated with human MPSs may further improve the accuracy of in vitro in vivo extrapolation for DDI studies by providing a human physiology-relevant experimental system (Mehta et al, 2024). The LTC platform also addresses the major challenges in the MPS field for absorption-distribution-metabolism-excretion applications (Fowler et al, 2020) by incorporating features such as medium recirculation, thermoplastic tissue chip material for low non-specific binding, sufficient volume for media-based sampling, high number of cells (ie, 215,000 per LTC) producing measurable metabolic outcomes, and low evaporation. The bioengineered millifluidic tissue chip enabled the long-term PK assessment of both low and high—clearance compounds, which is critical for assessing DDIs in vitro. While the focus of this study was on CYP3A4 substrates as victim drugs, further investigations will be necessary to assess the effect of induction on other CYP isoforms and non-CYP metabolizing enzymes and transporters. Additionally, assessing the perpetrator dose-response relationship will determine the maximum achievable induction for perpetrator drugs. Furthermore, evaluation of both perpetrator and victim drugs with various PHH donors will offer valuable insights to better understand the population variability in clinical DDI. While the LTC was initially validated with only PHH cells, future plans include the addition of human non-parenchymal cells to support broader applications such as disease modeling and metabolic studies, capturing more complex liver processes. Although the system may not offer the high-throughput capabilities of simpler in vitro models, its ability to generate detailed, high-content data from a single study makes it a valuable tool for lead optimization and preclinical drug discovery. Further, this platform serves as a foundation for future development of multiorgan interconnected systems that could better predict human responses (Edington et al, 2018; Wang et al, 2019) and provide physiologically relevant data before first-in-human studies (Rajan et al, 2023).

## Conflict of interest

Javelin Biotech authors are employees or former employees of Javelin Biotech Inc and may hold equity in Javelin Biotech. Murat Cirit is a cofounder, equity holder, and a board member.

## References

[bib1] Backman J.T., Kivistö K.T., Olkkola K.T., Neuvonen P.J. (1998). The area under the plasma concentration-time curve for oral midazolam is 400-fold larger during treatment with itraconazole than with rifampicin. Eur J Clin Pharmacol.

[bib2] Baudy A.R., Otieno M.A., Hewitt P., Gan J., Roth A., Keller D., Sura R., Van Vleet T.R., Proctor W.R. (2020). Liver microphysiological systems development guidelines for safety risk assessment in the pharmaceutical industry. Lab Chip.

[bib3] Bohnert T., Patel A., Templeton I., Chen Y., Lu C., Lai G., Leung L., Tse S., Einolf H.J., Wang Y.H. (2016). Evaluation of a new molecular entity as a victim of metabolic drug-drug interactions-an industry perspective. Drug Metab Dispos.

[bib4] Chan T.S., Yu H., Moore A., Khetani S.R., Tweedie D. (2019). Meeting the challenge of predicting hepatic clearance of compounds slowly metabolized by cytochrome P450 using a novel hepatocyte model, HepatoPac. Drug Metab Dispos.

[bib5] Dechanont S., Maphanta S., Butthum B., Kongkaew C. (2014). Hospital admissions/visits associated with drug-drug interactions: a systematic review and meta-analysis. Pharmacoepidemiol Drug Saf.

[bib6] Di L., Obach R.S. (2015). Addressing the challenges of low clearance in drug research. AAPS J.

[bib7] Du Y., Li N., Yang H., Luo C., Gong Y., Tong C., Gao Y., Lü S., Long M. (2017). Mimicking liver sinusoidal structures and functions using a 3D-configured microfluidic chip. Lab Chip.

[bib8] Edington C.D., Chen W.L.K., Geishecker E., Kassis T., Soenksen L.R., Bhushan B.M., Freake D., Kirschner J., Maass C., Tsamandouras N. (2018). Interconnected microphysiological systems for quantitative biology and pharmacology studies. Sci Rep.

[bib9] Fahmi O.A., Kish M., Boldt S., Obach R.S. (2010). Cytochrome P450 3A4 mRNA is a more reliable marker than CYP3A4 activity for detecting pregnane X receptor-activated induction of drug-metabolizing enzymes. Drug Metab Dispos.

[bib10] Fowler S., Chen W.L.K., Duignan D.B., Gupta A., Hariparsad N., Kenny J.R., Lai W.G., Liras J., Phillips J.A., Gan J. (2020). Microphysiological systems for ADME-related applications: current status and recommendations for system development and characterization. Lab Chip.

[bib11] Gorski J.C., Vannaprasaht S., Hamman M.A., Ambrosius W.T., Bruce M.A., Haehner-Daniels B., Hall S.D. (2003). The effect of age, sex, and rifampin administration on intestinal and hepatic cytochrome P450 3A activity. Clin Pharmacol Ther.

[bib12] Gu Q., Dillon C.F., Burt V.L. (2010). Prescription drug use continues to increase: U.S. Prescription drug data for 2007-2008. NCHS Data Brief.

[bib13] Herman R.J., Nakamura K., Wilkinson G.R., Wood A.J. (1983). Induction of propranolol metabolism by rifampicin. Br J Clin Pharmacol.

[bib14] Hultman I., Vedin C., Abrahamsson A., Winiwarter S., Darnell M. (2016). Use of HμREL human coculture system for prediction of intrinsic clearance and metabolite formation for slowly metabolized compounds. Mol Pharm.

[bib15] Imai H., Kotegawa T., Tsutsumi K., Morimoto T., Eshima N., Nakano S., Ohashi K. (2008). The recovery time-course of CYP3A after Induction by St John’s Wort Administration. Br J Clin Pharmacol.

[bib16] Kenny J.R., Ramsden D., Buckley D.B., Dallas S., Fung C., Mohutsky M., Einolf H.J., Chen L., Dekeyser J.G., Fitzgerald M. (2018). Considerations from the innovation and quality induction working group in response to drug-drug interaction guidances from regulatory agencies: focus on CYP3A4 mRNA in vitro response thresholds, variability, and clinical relevance. Drug Metab Dispos.

[bib17] Khetani S.R., Bhatia S.N. (2008). Microscale culture of human liver cells for drug development. Nat Biotechnol.

[bib18] Kidambi S., Yarmush R.S., Novik E., Chao P., Yarmush M.L., Nahmias Y. (2009). Oxygen-mediated enhancement of primary hepatocyte metabolism, functional polarization, gene expression, and drug clearance. Proc Natl Acad Sci U S A.

[bib19] Kratochwil N.A., Meille C., Fowler S., Klammers F., Ekiciler A., Molitor B., Simon S., Walter I., McGinnis C., Walther J. (2017). Metabolic profiling of human long-term liver models and hepatic clearance predictions from in vitro data using nonlinear mixed-effects modeling. AAPS J.

[bib20] Link B., Haschke M., Grignaschi N., Bodmer M., Aschmann Y.Z., Wenk M., Krähenbühl S. (2008). Pharmacokinetics of intravenous and oral midazolam in plasma and saliva in humans: usefulness of saliva as matrix for CYP3A phenotyping. Br J Clin Pharmacol.

[bib21] Lu C., Li A.P. (2001). Species comparison in P450 induction: effects of dexamethasone, omeprazole, and rifampin on P450 isoforms 1A and 3A in primary cultured hepatocytes from man, Sprague-Dawley rat, minipig, and beagle dog. Chem Biol Interact.

[bib22] Lu C., Di L. (2020). *In vitro* and *in vivo* methods to assess pharmacokinetic drug–drug interactions in drug discovery and development. Biopharm Drug Dispos.

[bib23] Mehta V., Karnam G., Madgula V. (2024). Liver-on-chips for drug discovery and development. Mater Today Bio.

[bib24] Pirmohamed M., James S., Meakin S., Green C., Scott A.K., Walley T.J., Farrar K., Park B.K., Breckenridge A.M. (2004). Adverse drug reactions as cause of admission to hospital: prospective analysis of 18 820 patients. BMJ.

[bib25] Prueksaritanont T., Kuo Y., Tang C., Li C., Qiu Y., Lu B., Strong-Basalyga K., Richards K., Carr B., Lin J.H. (2006). In vitro and in vivo CYP3A64 induction and inhibition studies in rhesus monkeys: a preclinical approach for CYP3A-mediated drug interaction studies. Drug Metab Dispos.

[bib26] Rajan S.A.P., Sherfey J., Ohri S., Nichols L., Smith J.T., Parekh P., Kadar E.P., Clark F., George B.T., Gregory L. (2023). A novel milli-fluidic Liver Tissue Chip with continuous recirculation for predictive pharmacokinetics applications. AAPS J.

[bib27] Reitman M.L., Chu X., Cai X., Yabut J., Venkatasubramanian R., Zajic S., Stone J.A., Ding Y., Witter R., Gibson C. (2011). Rifampin’s acute inhibitory and chronic inductive drug interactions: experimental and model-based approaches to drug–drug interaction trial design. Clin Pharmacol Ther.

[bib28] Steinbronn C., Yang X., Yu J., Dimova H., Huang S.M., Ragueneau-Majlessi I., Isoherranen N. (2021). Do inhibitory metabolites impact DDI risk assessment? Analysis of *in vitro* and *in vivo* data from NDA reviews between 2013 and 2018. Clin Pharmacol Ther.

[bib29] Templeton I.E., Chen Y., Mao J., Lin J., Yu H., Peters S., Shebley M., Varma M.V. (2016). Quantitative prediction of drug–drug interactions involving inhibitory metabolites in drug development: how can physiologically based pharmacokinetic modeling help?. CPT Pharmacometrics Syst Pharmacol.

[bib30] Tsamandouras N., Chen W.L.K., Edington C.D., Stokes C.L., Griffith L.G., Cirit M. (2017). Integrated gut and liver microphysiological systems for quantitative in vitro pharmacokinetic studies. AAPS J.

[bib31] van Dyk M., Marshall J.C., Sorich M.J., Wood L.S., Rowland A. (2018). Assessment of inter-racial variability in CYP3A4 activity and inducibility among healthy adult males of Caucasian and South Asian ancestries. Eur J Clin Pharmacol.

[bib32] Wang X., Cirit M., Wishnok J.S., Griffith L.G., Tannenbaum S.R. (2019). Analysis of an integrated human multiorgan microphysiological system for combined tolcapone metabolism and brain metabolomics. Anal Chem.

[bib33] Xie F., Ding X., Zhang Q.Y. (2016). An update on the role of intestinal cytochrome P450 enzymes in drug disposition. Acta Pharm Sin B.

[bib34] Xu Y., Zhou Y., Hayashi M., Shou M., Skiles G.L. (2011). Simulation of clinical drug-drug interactions from hepatocyte CYP3A4 induction data and its potential utility in trial designs. Drug Metab Dispos.

[bib35] Zanger U.M., Schwab M. (2013). Cytochrome P450 enzymes in drug metabolism: regulation of gene expression, enzyme activities, and impact of genetic variation. Pharmacol Ther.

[bib36] Zhu B., Liu Z.Q., Chen G.L., Chen X.P., Ou-Yang D.S., Wang L.S., Huang S.L., Tan Z.R., Zhou H.H. (2003). The distribution and gender difference of CYP3A activity in Chinese subjects. Br J Clin Pharmacol.

